# East meets west: integrating Yin-Yang theory with immunology teaching

**DOI:** 10.3389/fimmu.2024.1441863

**Published:** 2024-08-20

**Authors:** Zhiyong Wang, Min Wang, Mao Lin, Yanxin Lu, Qiang Xia, Pei Wei

**Affiliations:** ^1^ Department of Immunology, Zunyi Medical University, Zhuhai, China; ^2^ Department of Pharmaceutics, Zunyi Medical University, Zhuhai, China; ^3^ Department of Physiology, Zunyi Medical University, Zhuhai, China

**Keywords:** immune equilibrium, immunology teaching, Yin-Yang theory, gut microbiota, mucosal immunity

## Abstract

This perspective article delves into a novel integration of Yin-Yang theory—an ancient Chinese philosophical cornerstone—with the sophisticated realm of immunology. Given the intricate concepts inherent in immunology, many students find it challenging to comprehend the delicate mechanisms governing immune equilibrium and regulation. Given the deep-rooted understanding of Yin-Yang theory among Chinese students, we advocate for an educational strategy that contextualizes the concept of immune equilibrium within the framework of Yin-Yang, thereby offering a more intuitive and engaging learning experience. This method not only capitalizes on the cultural significance of Yin-Yang, but also corresponds to its principles of equilibrium and harmony, thus mirroring the homeostatic essence of immune responses. This article critically assesses this technique’s capacity to bolster immune comprehension amongst Chinese students, while also considering its limitations. Despite these limitations, the fusion of these seemingly divergent fields holds substantial promise for augmenting immunology education, promoting critical thinking, and advancing cross-cultural academic discourse. The amalgamation of age-old philosophical insights with modern scientific exploration prompts a reassessment of educational methodologies within immunology, underscoring a novel pedagogical approach that bridges traditional wisdom with contemporary scientific education.

## Introduction

Immunology, a pivotal field within medical science, unveils the complex mechanisms that shield organisms from infection and disease. Its depth, characterized by the sophisticated interplay of cellular and molecular processes, presents considerable challenges for learners, especially undergraduates at the outset of their scientific exploration. Among these challenges is the concept of immune equilibrium, which emerges as a significant hurdle. This requires students to untangle the web of regulatory mechanisms that preserve health and thwart disease.

The philosophy of Yin and Yang, which is rooted in ancient Chinese dialectical thinking, symbolizes a state in which contradiction and harmony coexist. It mirrors the concept of dynamic equilibrium in immunology—the intricate equilibrium between immune response and tolerance. Within the cultural milieu of China, Yin-Yang principles are not esoteric concepts, but integral parts of everyday thought. This cultural familiarity offers a rich base for employing Yin-Yang theory as a pedagogical tool in immunology education. By drawing analogies between the dialectical nature of immune responses—such as the equilibrium between pro-inflammatory and anti-inflammatory signals, or the regulation between immune activation and suppression—we can distill complex ideas into more comprehensible segments of knowledge.

This perspective article aims to investigate the potential role of Yin-Yang philosophy in immunology education. Specifically, it attempts to demonstrate how this age-old wisdom can deepen our understanding of immune equilibrium, simplify the learning journey, and narrow the divide between intricate scientific notions and their intuitive understanding. Concurrently, it scrutinizes the distinctions between the abstract philosophy of Yin-Yang, and the empirical foundations of immunology. This ensures that when merging such traditional insights with contemporary scientific teaching, the accuracy and thoroughness of the latter remain intact.

## Immune equilibrium: a paradigm of dialectical harmony in immunology

While traditionally seen as distinct fields, immunology and philosophy are intertwined through continuous dialogue between immunologists and philosophers. This cross-disciplinary engagement enriches both fields—immunologists gain philosophical insights into the workings of the immune system, while philosophers explore the essence of self and life through the lens of immunology. Such collaborations underline the value of cross-field knowledge exchange.

A fundamental concept within immunology is the system’s capacity to distinguish between benign antigens and healthy tissue, versus harmful pathogens. Various theories have been developed, including concepts of self- and non-self-recognition (1–3), infectious non-self and noninfectious self-recognition (4), danger-recognition (5), microbial patterns and abnormal self-markers-recognition (6–8), antigenic discontinuity recognition (9), and the more complex immune equilibrium theory (10–13), among others. Immune equilibrium, with its philosophical roots, presents a unique challenge for students unfamiliar with philosophical thought.

The concept of immune equilibrium traces back to the seminal work of scientist Elias Metchnikoff, whose pioneering study of phagocytic cells laid the foundation for this theory. Metchnikoff introduced the idea that phagocytes continuously discern between self- and non-self-entities (14, 15). He proposed that the symbiotic relationship between microbes and the human body emerges from a balanced contention between these microorganisms and the phagocytic cells (14, 15). This interpretation bridged the immunological and philosophical perspectives. Macfarlane Burnet—a key figure in the development of immune recognition theory—further refined this concept. Burnet offered deep insights into the immunological scheme of self- and non-self-recognition, and the clonal selection of antibodies (16), thereby significantly impacting the field. He viewed immunology as much a philosophical inquiry as a scientific one, suggesting an inherent drive within both microbial and immune cells towards establishing an ecological equilibrium. He therefore introduced a mechanism of dynamic equilibrium, where interactions between immune and non-immune cells are regulated by modulating cell quantity and composition (10, 17). Building on this theoretical framework, Bartlomiej Swiatczak systematically summarized the theory of immune equilibrium (10). Furthermore, Gérard Eberl outlined a specific cellular-level model of immune equilibrium, dependent on a dynamic equilibrium among four competing and mutually inhibitory immune responses (11). Moreover, subsequent analysis by Joseph M. Cicchese and others delved into how the dynamic modulation of pro-inflammatory and anti-inflammatory signals maintains immune equilibrium (12). More recently, Shevyrev et al. have suggested that immune equilibrium relies heavily on the interplay between antigen presentation and recognition, further bridging the gap between immune recognition and equilibrium theories (13). These diverse perspectives converge on a crucial theme—immune function cannot be simplistically categorized into binary oppositions like self- and non-self-recognition, or activation/inactivation states. Rather, a healthy immune system is characterized by its perpetual activity, and its ability to maintain a dynamic equilibrium between promoting and antagonistic responses (**Table 1**). This equilibrium is co-regulated by a complex interplay among immune cells, immune molecules, and the microbial environment.

**Table 1:** Summary of key perspectives on immune equilibrium.

Author(s)	key perspectives	References
Elie Metchnikoff	The author demonstrated the existence of phagocytes and pointed out that the symbiotic relationship between microbes and their hosts emerges from a balanced contention between these microorganisms and the phagocytes.	(14, 15)
Theobald Smith	The author believed that, in the evolutionary process of parasite-host interactions, the two parties gradually tend towards equilibrium, and infectious diseases represented a manifestation of the imbalance in the struggle between parasites and hosts.	(40)
Hans Zinsser	The author believed that the core of the mutual adaptation between parasites and hosts lies in the balance of their antagonistic interactions.	(41)
Felix d’Herelle	The author, through research on bacteriophages, demonstrated that both pathogens and hosts have significant abilities to adapt to each other’s increasing virulence and resistance, eventually leading to a perfectly balanced interaction between them.	(42, 43)
Niels Jerne	The author believed that the fundamental role of the immune system was to restore the disrupted balance by generating an appropriate antibody response.	(44)
Macfarlane Burnet	The author identified an intrinsic tendency in both microbial and immune cells to seek ecological balance. Thus, he proposed a dynamic equilibrium mechanism, regulating interactions between immune and non-immune cells by adjusting cell quantity and composition.	(10, 17)
Bartlomiej Swiatczak	The author systematically summarized the theory of immune balance, proposing that the immune system is not a killer of non-self entities, but rather a peacemaker that helps establish harmony with the environment.	(10)
Gérard Eberl	The author proposed that the immune system maintains a dynamic balance among four mutually inhibitory responses: Type 1 (targeting intracellular threats like viruses), Type 2 (addressing large parasites), Type 3 (focusing on extracellular microorganisms), and Type 4 (protecting sensitive tissues by secreting substances such as IgA).	(11)
Joseph M. Cicchese et al.	The authors believed that the dynamics of pro- and anti-inflammatory immune responses influence disease progression. These responses can lead to outcomes such as high pathogen burden, severe tissue damage, cleared infection with restored inflammation levels, or a balanced response that controls pathogen growth and limits host damage.	(12)
Daniil Shevyrev et al.	The authors suggested that immune system equilibrium is dynamically maintained by genetic HLA variants, which shape TCR repertoires and individualize immune responses. Disruptions in this balance, leading to various diseases, are associated with changes in antigen presentation, T- and B-cell repertoires, and cell specialization.	(13)

In essence, the theory of immunological equilibrium describes various layers of balance, including the ecological harmony between hosts and microbes, the interplay among different types of immune cells, and the interactions within immune molecules. This complexity means that immunology does not follow a simple linear knowledge structure; rather, it is networked, linking each concept to multiple other concepts. Such interconnectedness makes immunology one of the more challenging subjects to master in the realm of medical science. Therefore, incorporating a philosophical perspective into the teaching of immunology could be highly beneficial. Given that most students may not have extensive experience with philosophical reasoning, leveraging philosophically rich concepts from everyday experiences to elucidate immunological equilibrium might significantly enhance learning outcomes.

## Equilibrium and harmony: the core concepts of Yin-Yang theory

**Figure 1:**
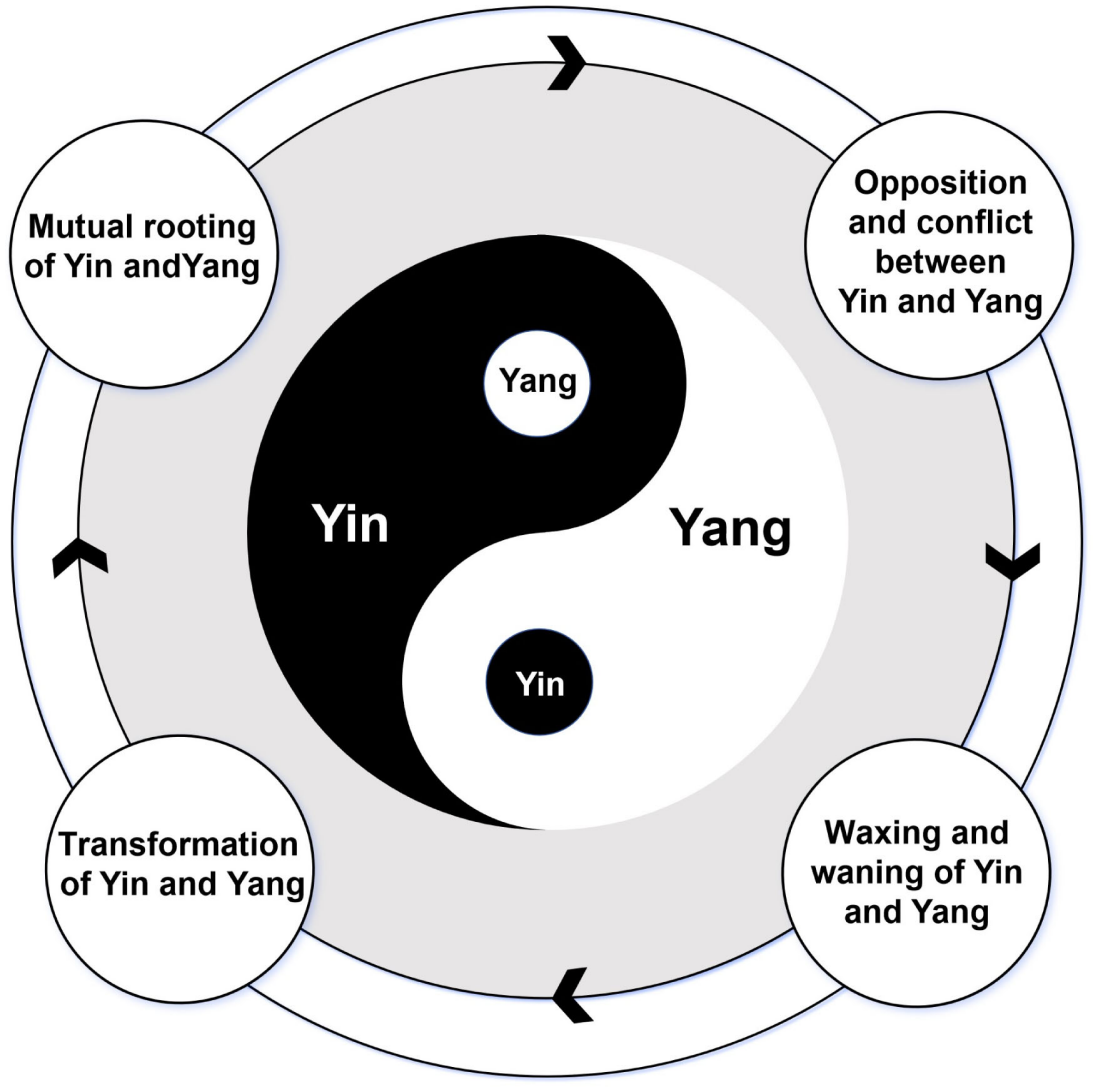
Illustration of the four key principles of Yin-Yang theory. This figure visually represents the four fundamental principles of the Yin-Yang theory in traditional Chinese philosophy. Mutual rooting of Yin andYang: Yin and Yang are mutually dependent, with each being a condition for the other’s existence. Opposition and conflict between Yin and Yang: Yin and Yang are opposites that conflict with one another, but through their dynamic interactions, they tend toward equilibrium. Waxing and waning of Yin and Yang: Yin and Yang are in constant flux, experiencing periods of increase and decrease, which ensures a cyclical balance that reflects natural order. Transformation of Yin and Yang: Under certain conditions, Yin and Yang can transform into each other, emphasizing their fluidity and mutual permeability, wherein each contains the seed of its opposite (Yin within Yang and Yang within Yin).

The Yin-Yang theory—a foundational element of traditional Chinese philosophy—offers a sophisticated framework to understand the dynamic interplay of forces in nature and human health (18–20). This theory is articulated through four key principles that encapsulate the intricate relationship between Yin (the passive, cooling, and downward-moving elements) and Yang (the active, warming, and upward-moving elements). The first principle is the mutual rooting of Yin and Yang, which emphasizes the co-dependency of the two elements. Their existence is mutually dependent, highlighting an ecological and physiological interconnectedness, where each force is a condition for, and a premise of the other’s presence. Second, the opposition and conflict between Yin and Yang is as follows: Despite their inherent contradictions, Yin and Yang form a unified whole. Their interaction fosters a dynamic equilibrium, which is crucial for maintaining normal physiological functions, and overall harmony. Disruptions to this equilibrium can lead to illness, illustrating the importance of their conflict and interdependence. Third, Yin and Yang are characterized by a constant flux, or waxing and waning. That is, each undergoes periods of dominance and recession. This ensures a relative equilibrium, with each phase of growth or decline leading to the next, embodying the cyclical nature of these forces. Last, Yin and Yang can transform into one another under certain conditions, emphasizing the fluidity and interchangeability of these energies. This transformation is not an instantaneous event but a dynamic process. A key state within this process is the presence of elements of Yang within Yin, and elements of Yin within Yang. This interpenetration underscores the complexity and interconnectedness of these dual forces, emphasizing that within each polarity lies the seed of its opposite (**Figure 1**).

At its heart, the Yin-Yang theory presents a cosmological view of the universe as governed by complementary yet opposing forces. These forces, which are constantly seeking equilibrium and harmony, are emblematic of everything, displaying characteristics that are opposite but interdependent. The notion that each element harbors the potential for its opposite illustrates the dialectical process of interaction leading to transformation, capturing the essence of dialectical thinking.

Ultimately, the Yin-Yang theory provides a profound philosophical framework that highlights the significance of equilibrium, interconnectivity, and transformation in the natural order. This model sheds light on traditional Chinese thought, while also offering a universally relevant lens to comprehend the complexities of immune equilibrium.

## Bridging Yin-Yang theory and immune equilibrium: a case study on gut mucosal immunity

To integrate the concept of Yin-Yang theory into the teaching of immunology, we focus on a case study of the gut immune system. The mucosal surface of the gut is inhabited by a microbial community, including bacteria, archaea, fungi, viruses, and parasites, which has co-evolved with the mammalian host immune system. Beyond digestion and nutrient absorption, these microbes are crucial for the normal functioning of mucosal immunity. In a sense, these commensal microbes should also be considered a special component of the immune system, given their vital role in maintaining gut immune homeostasis (21, 22). However, it should be recognized that this discussion will not delve into highly specific immunological regulatory mechanisms. As famously stated, “Education is not the filling of a pail, but the lighting of a fire.” Therefore, we aim to explain how students can use the doctrine of Yin-Yang to recognize, understand, think about, and question the phenomena and mechanisms included in the theory of immune equilibrium.

**Figure 2:**
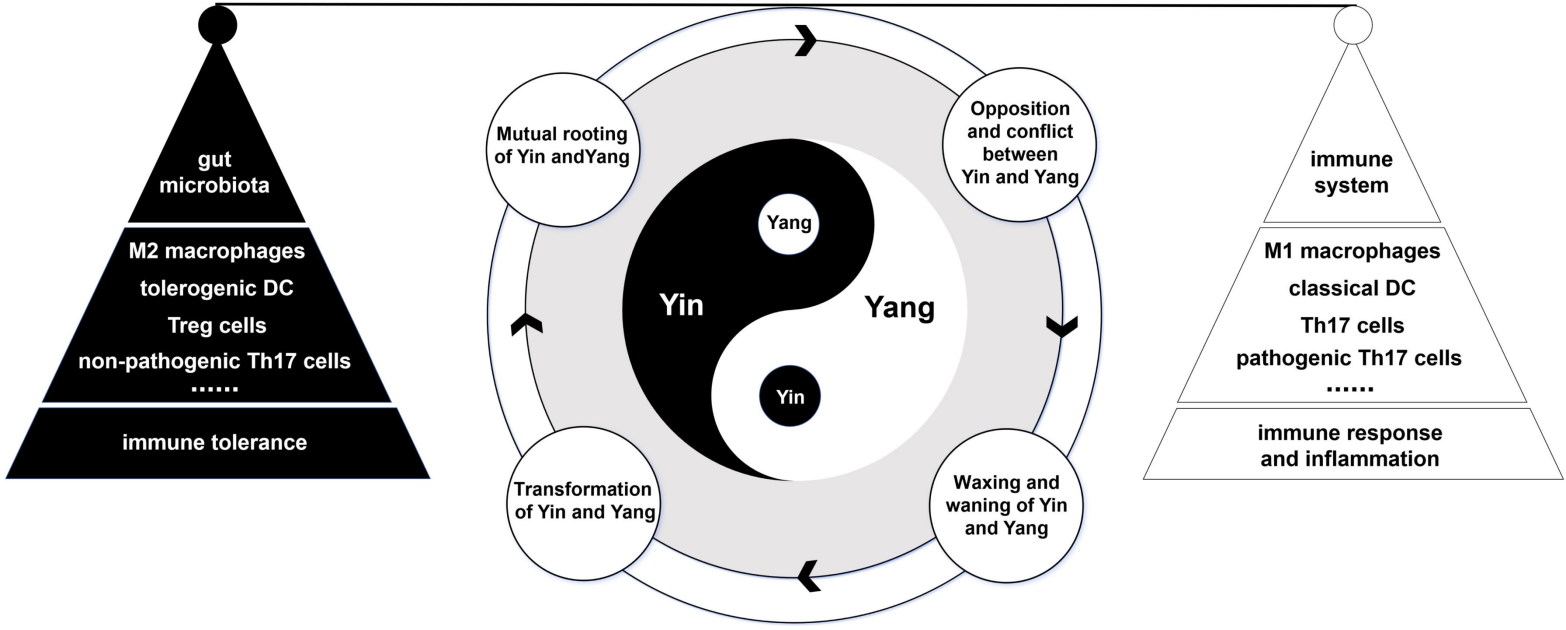
The conceptual framework of Yin-Yang theory in gut mucosal immune equilibrium. The application of the Yin-Yang theory to the mucosal immune equilibrium in the gut can be explored from three perspectives: the interaction between the host immune system and the gut microbiota, the interplay of cells and molecules within the immune system itself, and the overall immune equilibrium of the host organism. In each perspective, a deeper understanding of the concept of immune equilibrium can be achieved through the lens of the four principles of the Yin-Yang theory. The cyclical operation of these Yin-Yang principles corresponds to the dynamic changes in immune equilibrium.

The concept of immune equilibrium should be approached from at least three distinct levels, including: the equilibrium between the host immune system and environmental entities, such as gut microbiota; the interplay of cells and molecules within the immune system itself; and the overall immune equilibrium of the host organism. Whether one considers the equilibrium within these individual levels or the overarching immune homeostasis, the enduring principles of the Four Elements theory of Yin-Yang offer profound insights (**Figure 2**).

When exploring the immune equilibrium between the host immune system and environmental components—such as in the mucosal immunology of the gut—it is critical for educators to convey that the immune system and gut microbiota should not be viewed in isolation, but rather as two integral parts of a unified entity. By conceptualizing the immune system as “Yang” and the gut microbiota as “Yin”, we can gain deeper insights into their interactions through the lens of the Four Elements theory. First, the principle of the “Mutual rooting of Yin and Yang” elucidates the symbiotic relationship between the immune system and the gut microbiota, highlighting their mutual benefits and lifelong impact. The immune system regulates the composition and function of the symbiotic microbial communities, and these microbes influence immune development and function, initiating immune responses through their metabolic products or structural components. In this regard, the presence of antigen-specific immune cells in a steady state serves as an exemplary model. It not only aids in elucidating the role of the gut microbiota in the development and maturation of immune cell populations but also underscores the critical importance of immune training and development acquired during immune system maturation for achieving immune tolerance to “commensal” microbiota. For instance, bacteria-specific naive B cells and memory B cells can be detected not only in the gut but also in systemic immune compartments such as the spleen (23, 24). Second, according to the principle of “opposition and conflict between Yin and Yang,” although the gut microbiota and the immune system achieve a state of equilibrium through their interactions, this balance is inherently fragile. The gut, as the organ with the largest surface area in contact with the external environment, undergoes constant changes in its microbial composition. The introduction of new pathogenic microorganisms can disrupt the existing microbial community, potentially breaking this immune equilibrium and leading to a state of Yin-Yang imbalance characterized by opposition and conflict. This dynamic process, while potentially intense, ultimately aims to re-establish a new balance between the microbiota and the immune system. A quintessential example of this process is the reconstruction of the gut microbiota following intestinal inflammation. Third, the principle of the ‘waxing and waning of Yin and Yang’ emphasizes the dynamic interplay between the gut microbiota and the immune system in achieving immunological equilibrium. This dynamic interaction not only reflects the regulatory influence of microbes on the immune system but also the immune system’s process of accepting and integrating new microbial entities. A pertinent example is the role of maternal IgA in modulating the symbiotic colonization of the neonatal gut microbiota (25, 26). For instance, the introduction of Bacteroides thetaiotaomicron into germ-free mice can elicit an immune response. However, IgA targeting the capsular polysaccharide 4 (CPS4) of Bacteroides thetaiotaomicron can inhibit CPS4 synthesis and induce CPS5 synthesis, thereby promoting immune tolerance to Bacteroides thetaiotaomicron (26). Finally, the principle of the “transformation of Yin and Yang” implies that in their struggle and opposition, one aspect may become part of the other. For example, IgA not only participates in the clearance of gut bacteria, but may also facilitate their retention and maintenance by becoming partly incorporated into the bacteria (IgA encapsulating bacteria) (27, 28). For gut microbiota, one way to integrate into the immune system is by inducing the development of immune cells and modulating immune responses through secreted metabolic products (29, 30). Similarly, we can illustrate this principle from different perspectives, such as within the context of the same disease, system, or type of immune cell. These perspectives are also applicable to other principles of the Yin-Yang theory. When explaining immune equilibrium through the lens of Yin-Yang theory, teachers should seize opportunities to inspire students to think analogously, thereby deepening their understanding of immunological concepts.

At the cellular and molecular levels, gut mucosal immunity embodies a dynamic equilibrium and unity of opposites, aligning seamlessly with the four principles of the Yin-Yang theory. Among the many cells and molecules involved in gut mucosal immunity, there are several representative examples, including: the equilibrium between Th1 and Th2 cells and their regulators, the equilibrium between M1 and M2 macrophages and their regulators, the interplay between Th17 and Treg cells and their regulators, and the interplay between pathogenic and non-pathogenic Th17 cells and their regulatory factors. Taking pathogenic and non-pathogenic Th17 cells as an example, if we consider non-pathogenic Th17 cells as “Yin” and pathogenic Th17 cells as “Yang,” the four principles of Yin-Yang theory offer a valuable framework for understanding their relationship. First, according to the principle of “Mutual rooting of Yin and Yang,” both pathogenic and non-pathogenic Th17 cells originate from naive T cells and share developmental pathways. Differentiation outcomes depend on the cytokine environment surrounding naive T cells. For instance, IL-6/IL-1β/IL-23 or IL-6/TGF-β3 can induce pathogenic Th17 cells (31, 32), while IL-6 and TGF-β1 induce non-pathogenic Th17 cells (31, 33). Furthermore, the principle of “opposition and conflict between Yin and Yang” dictates that pathogenic Th17 cells are often associated with inflammatory immune pathology (34–36), whereas non-pathogenic Th17 cells express anti-inflammatory cytokines, such as IL-10, which can suppress inflammation and limit host tissue damage (37, 38). Thus, they exist in a state of mutual antagonism within the immune response framework. Given the variations in cytokine secretion profiles during inflammation, the differentiation of pathogenic and non-pathogenic Th17 cells is undoubtedly subject to dynamic fluctuations, consistent with the principle of the waxing and waning of Yin and Yang. Lastly, the principle of “transformation of Yin and Yang” implies that pathogenic and non-pathogenic Th17 cells can transform into one another due to shifts in the immune microenvironment. The interconversion between pathogenic and non-pathogenic Th17 cells is influenced partly by changes in cytokine levels in the environment (31–33), and partly by the regulation of other immune molecules. For example, the absence of CD5L can convert non-pathogenic Th17 cells into pathogenic Th17 cells (39). In summary, viewing these cellular dynamics through the lens of Yin-Yang theory provides a nuanced understanding of how the balance between pathogenic and non-pathogenic Th17 cells is maintained, and how its disruption may lead to disease.

As mentioned, immune equilibrium constitutes an integration of balances at various levels, making a comprehensive understanding of holistic immune equilibrium within hosts imperative, particularly the equilibrium between immune response and immune tolerance. Essentially, this equilibrium represents a state where the immune system adjusts and responds to environmental changes, achieving immune tolerance to mitigate potential deleterious effects. This process can guide students to comprehend that the ultimate goal of immune responses is to establish mutual immune tolerance between the individual and the environment, predicated on the continuous activation of the immune system. From this perspective, immune response and immune tolerance are interdependent, embodying the principle of mutual containment seen in the “Mutual rooting of Yin and Yang”. The transition from immune response to immune tolerance involves a dynamic process of opposition and fluctuation, where each aspect alternatively suppresses and enhances the other. This reflects the principles of “opposition and conflict between Yin and Yang”, and “waxing and waning of Yin and Yang”. When a dynamic equilibrium between immune response and immune tolerance is achieved, shifts from commensal microorganisms to opportunistic pathogens can transform elements of immune tolerance into participants in the immune response, and vice versa. This transformation encapsulates the principle of the “transformation of Yin and Yang” within the host’s immune equilibrium dynamics.

Viewing the gut mucosal immune equilibrium through the collective lens of these three levels—underpinned by the four principles of Yin-Yang theory—enables a comprehensive understanding that encompasses the ecological complexity of the gut environment. This approach underscores the importance of harmony and equilibrium across all dimensions of immune interactions, from the macroscopic ecosystem inhabited by the host and its microbiome, to the microscopic world of cellular and molecular dynamics. By applying Yin-Yang theory holistically, one appreciates the fluidity of immune equilibrium, recognizing that health represents a state of dynamic equilibrium rather than a static condition. Such an integrated perspective not only enriches our conceptual grasp of gut immunity, but also highlights the potential for interventions that restore balance through modulating either side of these interrelated systems.

Incorporating the philosophy of Yin and Yang into the teaching framework of immune equilibrium offers a distinctive advantage by bridging the gap between traditional cultural wisdom and contemporary scientific understanding, especially for Chinese students. This integration enriches the educational experience by framing the complex principles of immune equilibrium within the familiar context of Yin-Yang theory, thereby enhancing cognitive engagement among students. Utilizing the profound insights of Yin-Yang theory―including mutual dependency, oppositional struggle, dynamic equilibrium, and the potential for cyclical transformation―encourages students to adopt a holistic, developmental, and dialectical perspective in their study of immunology. This interdisciplinary fusion not only bridges the cultural and scientific divide but, more importantly fosters a learning environment that values diversity in thought and methodology.

## Challenges in integrating Yin-Yang theory into immunology teaching

Integrating the philosophy of Yin-Yang into the teaching of immunology offers a pioneering approach that marries traditional wisdom with modern scientific concepts, particularly resonating with students who have a Chinese cultural background. Despite its promise, this approach is laden with challenges, including potential epistemological conflicts, the simplification of complex scientific ideas, the unintended elevation of non-empirical reasoning, and concerns about cultural inclusivity.

First, while the Yin-Yang theory offers a compelling framework for understanding the dynamic balance within biological systems, not all aspects of immunology can be seamlessly integrated into this philosophical paradigm. For instance, certain characteristics exhibited by immune mechanisms do not fit neatly into these two categories, complicating the application of the Yin-Yang theory. This discrepancy underscores the need for nuanced interpretations rather than forcing every immunological phenomenon to align with Yin-Yang principles. Particularly, the attempt to apply the binary structure of Yin and Yang to immune equilibrium could inadvertently lead to an oversimplified view of the immune system’s complexity. With its myriad interactions between cells, molecules, and signaling pathways, reducing the immune system’s processes to a simple dichotomy might lead to misrepresentations and misunderstandings about the nature of scientific exploration. Therefore, when applying the Yin-Yang framework to teach immunology, educators should selectively highlight examples where this perspective provides valuable insights, while avoiding overextending analogies into areas where they may not be applicable. This selective approach ensures that traditional wisdom is respected and appropriately utilized without compromising the scientific integrity of immunological concepts. In addition, educators must clearly emphasize to students that the Yin-Yang theory serves as an illustrative tool rather than a universal solution, thereby fostering critical thinking throughout the learning process.

Moreover, the foundational approaches of the Yin-Yang philosophy and modern immunology diverge significantly. Where Yin-Yang embraces a holistic perspective focusing on equilibrium and interconnectedness through qualitative analysis, modern immunology is anchored in empirical research, emphasizing quantitative data and mechanistic explanations. This divergence raises the challenge of translating abstract Yin-Yang principles into the precise, data-driven language typical of immunological science.

Furthermore, leaning too heavily on the Yin-Yang concept to explain immune functions might prioritize the idea of equilibrium over rigorous empirical investigation and critical analysis. While the notion of equilibrium is undoubtedly relevant to understanding homeostasis and immune processes, an excessive focus on harmony could detract from examining pathological states or abnormal immune responses that do not align neatly with this conceptual framework.

Lastly, incorporating Yin-Yang philosophy into immunology education must be approached with sensitivity to avoid cultural bias. Although this methodology resonates strongly with students familiar with Chinese philosophical traditions, it may not engage or be as accessible to those from different backgrounds. This consideration underlines the importance of inclusivity and the risk of unintentionally sidelining students who do not share this cultural connection.

In summary, weaving Yin-Yang philosophy into immune education is an novel means to bridge cultural and scientific knowledge. Although promising, this approach is also fraught with numerous challenges. From the philosophical perspective, one can often find evidence that fits the philosophy, but not every piece of knowledge contains the full spectrum to fill the philosophical framework. From the scientific perspective, immunology is a relatively young subject and its knowledge is still evolving. This means that our understanding of immune mechanisms is continually being updated, and new discoveries can sometimes overturn established theories. Addressing these issues necessitates a balanced and thoughtful strategy that honors both traditional philosophical insights and the demands of rigorous scientific inquiry. This ensures that such integrative efforts clarify the intricacies of immunology, rather than obscure them.

## Conclusion

In conclusion, the integration of Yin-Yang philosophy into the teaching of immune equilibrium represents an innovative interdisciplinary approach that seeks to merge traditional Chinese philosophical wisdom with the empirical rigor of modern science. This approach, while demonstrating potential for a more holistic understanding of immunological processes, is fraught with challenges that stem from fundamental epistemological differences, the risk of oversimplification, and concerns regarding cultural inclusivity and bias. Moving forward, it becomes evident that the path to effectively incorporate traditional wisdom—such as Yin-Yang philosophy—into scientific education, requires a delicate equilibrium. This equilibrium must foster respect for cultural heritage and philosophical insights, while consistently adhering to the empirical evidence and critical thinking that propel scientific advancement. Such endeavors will undoubtedly enrich the educational landscape. However, they must be pursued with thoughtful consideration of their implications for both scientific understanding and cultural respect.

## Data Availability

The original contributions presented in the study are included in the article/Supplementary Material. Further inquiries can be directed to the corresponding authors.
